# TRIM37 promotes gallbladder cancer proliferation by activating the Wnt/β-catenin pathway via ubiquitination of Axin1

**DOI:** 10.1016/j.tranon.2023.101732

**Published:** 2023-06-26

**Authors:** Ming Xu, Bowen Jiang, Zhongran Man, Hongyi Zhu

**Affiliations:** a Department of Hepatobiliary Surgery, The First Affiliated Hospital of Bengbu Medical College, Bengbu, Anhui, China; b Department of Biliary-Pancreatic Surgery, Renji Hospital, School of Medicine, Shanghai Jiao Tong University, Shanghai, China

**Keywords:** Gallbladder cancer, Prognosis, TRIM37, Axin1, Wnt/β-catenin

## Abstract

**Background:**

Gallbladder cancer (GBC) is among the most lethal malignancies in the world, with a prognosis that is extremely poor. The results of previous studies suggest that tripartite motif containing 37 (TRIM37) contributes to the progression of numerous types of cancer. Nevertheless, there is little knowledge about the molecular mechanisms and functions of TRIM37 in GBC.

**Methods:**

A clinical significance assessment was conducted on TRIM37 following its detection by immunohistochemistry. In vitro and in vivo functional assays were performed to investigate the role of TRIM37 in GBC.

**Results:**

In this study, TRIM37 is upregulated in GBC tissues, which is associated with decreased histological differentiation, advanced TNM stage, and shorter overall survival rates. In vitro, TRIM37 knockdown inhibited cell proliferation and promoted apoptosis, and in vivo, TRIM37 knockdown suppressed GBC growth. Contrary to this, cell proliferation is increased in GBC cells when overexpression of TRIM37 is expressed. Mechanistic investigations revealed that TRIM37 promotes GBC progression through activation of the Wnt/β‑catenin signaling pathway via degradation of Axin1.

**Conclusion:**

The present study suggests that TRIM37 contributes to the development of GBC and thus provides an important biomarker for predicting GBC prognosis and an effective target for therapeutic intervention.

## Highlights

• The expression of TRIM37 was significantly higher in GBC tissues and associated with poor prognosis of GBC patients.
• TRIM37 promotes GBC development by activating Wnt/β-catenin signaling through the degradation of Axin1.
• TRIM37 contributes to the development of GBC and provides an important biomarker for predicting GBC prognosis and an effective target for therapeutic intervention.

## Graphical abstract

**Figure:**
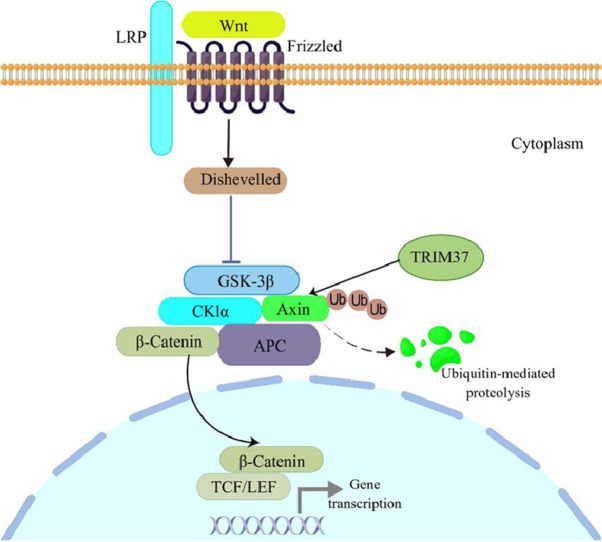

## Introduction

Among the biliary system tumors, gallbladder cancer (GBC) is the most common and most aggressive with an extremely poor prognosis [1]. For the treatment of GBC, surgical resection remains the most effective method at the moment. However, the lack of a specific early diagnostic method results in the majority of GBC patients being diagnosed at an advanced stage of the disease when patients lost the opportunity to receive radical resection [2,3]. In addition, GBC is often resistant to adjuvant treatments (chemotherapy and radiotherapy) [4]. Despite advances in GBC treatment, patients are most likely to develop recurrence or metastatic disease on a local or distant level with a poor prognosis and a survival rate of less than 10 percent after 5 years. For this reason, it is essential to elucidate the underlying molecular mechanisms that contribute to GBC progression and to develop more effective therapeutic treatments that improve prognosis.

Cancer progression may be caused by abnormal activation of the Wnt/β-catenin pathway in several cancer types, such as GBC [5,6]. Wnt ligands act to stabilize cytoplasmic β-catenin by inhibiting degradation by the Axin complex. In the role of cancer-suppressing agents, Axin1 is a crucial component of the canonical Wnt signaling pathway, and it acts as a scaffold for β-catenin degradation. Upon stimulation with Wnt, the degradation complex dissociates and β-catenin degradation is prevented. Cytoplasmic accumulation of β-catenin causes it to be translocated to the nucleus, which promotes cellular proliferation by up-regulating C-Myc, Cyclin D1, and other genes [7,8].

Tripartite motif-containing 37 (TRIM37) belongs to the tripartite motif (TRIM) family and displays a conserved RING finger, B-box, and coiled coil regions [9]. The RING finger domain of TRIM37, which is characteristic of most proteins in the TRIM family, enables it to act as an E3 ubiquitin ligase. Upregulated expression of TRIM37 plays a critical role in cancer progression and development. There has been research suggesting that there are several signaling pathways through which TRIM37 promotes tumor progression, including the Wnt/β-catenin pathway [10], [11], [12]. TRIM37 appears to play a role in GBC, but the mechanism remains unclear. As found in the current study, TRIM37 expression was upregulated in GBC tissues, which was associated with poor prognosis for patients. Investigations of biological functions indicate that the expression of TRIM37 promotes GBC cell proliferation. In addition, further mechanism studies suggest that TRIM37 could activate the Wnt/β-catenin signaling pathway via the degradation of Axin1.

## Materials and methods

### Patients and tissue specimens

The immunohistochemistry specimens used for immunohistochemistry were obtained from 124 radical cholecystectomy patients who underwent no preoperative chemotherapy or radiotherapy from January 2008 to January 2017 at the Department of Hepatobiliary Surgery, The First Affiliated Hospital of Bengbu Medical College. As shown in Table 1, the clinical and pathological characteristics of these 124 GBC patients were detailed. From an independent group of 10 GBC patients, fresh tissue samples and non-tumor tissue samples were collected for quantitative real-time PCR (qRT-PCR) and Western Blotting analysis from February 2019 to March 2020 and after removal, they should be stored at −80°C within 15 minutes. We received approval from our hospital's Ethics Committee for this project and informed consent was obtained from each patient enrolled before sample collection.

**Table 1:** Association between TRIM37 expression and clinicopathology feature in patients with GBC.

Characteristics	Cases	TRIM37 expression		χ^2^-value	*P*-value
		Low	High		
Age (years)					
<60	37	18	19	0.024	0.877
≥60	87	41	46		
Sex					
Male	39	21	18	0.895	0.344
Female	85	38	47		
Tumor size (cm)					
<3	58	29	29	0.256	0.613
≥3	66	30	36		
Tumor differentiation					
Well/moderate	59	35	24	6.221	0.013
Poor	65	24	41		
Lymph node metastasis					
No	68	33	35	0.054	0.816
Yes	56	26	30		
Liver metastasis					
No	59	33	26	3.148	0.076
Yes	65	26	39		
TNM stage					
I/II	40	26	14	9.784	0.020
III/IV	84	33	51		

p-values represent Pearson χ2–test. Bold values indicate statistical significance, P < 0.05.

### Immunohistochemistry

Paraffin-embedded GBC tissues were sectioned for immunohistochemical (IHC) analysis. Deparaffinization in xylene and rehydration in ethanol with varying concentrations were performed on the sections. In accordance with the standard procedure, the specimens were stained with an anti-TRIM37 antibody (Proteintech; 1:100 dilution) followed by an anti-IgG antibody A0208 (Beyotime, China) coupled to horseradish peroxidase (HRP). A maximum of four points was given to each sample in the immunohistochemistry evaluation, which was conducted independently by two pathologists without any knowledge of the patient's clinical or pathological characteristics. In total, 124 GBC patients were divided into two groups according to their IHC results: low TRIM37 expression group (0–2) and high TRIM37 expression group (3–4).

### Cell lines, cell culture and construction of lentiviral transfected cells

Genechem (Genechem Incorporation, Shanghai, China) provided the human GBC cell lines GBC-SD and NOZ on October, 2019. An analysis of short tandem repeats has been performed on the GBC-SD and NOZ cells to validate their authenticity. The cells used in this study were regularly tested for contamination with mycoplasma. GBC‐SD cells were cultured in high‐glucose DMEM (Hyclone, USA), and NOZ cells were cultured in William's medium E (Gibco, USA). In addition to 10% fetal bovine serum (Gibco, USA), 1% antibiotics were added to the medium to maintain all of the cells at 37°C in a humidified atmosphere with 5% CO2. From Selleck, we obtained cycloheximide (CHX) and MG132, which inhibit protein synthesis and proteasome activity, respectively.

The TRIM37-overexpressed and TRIM37-short-hairpin RNA (shTRIM37) lentiviruses, as well as their negative controls, were obtained from Genechem (Shanghai, China). The guiding strand site and sequences of shTRIM37, according to the literature report [13],were as follows: shTRIM37, position 1799-1817, 5′-GGAGAAGATTCAGAATGAA-3′. The indicated lentivirus was infected with GBC cells followed by puromycin selection over 7 days at 1.5 g/mL. QRT-PCR and Western blotting were used to determine the level of TRIM37 expression in the infected cells.

### Cell proliferation, colony formation and apoptosis assay

In order to perform the subsequent treatment, the transfected cells are plated on either six or 96-well plates, and they are cultured for the indicated period of time. An assay for evaluating cell proliferation was measured using the Cell Counting Kit-8 (CCK-8, Bimake, China). A cellular colony assay was performed by plating 1000 cells per well in 6-well plates and culturing them for two weeks at 37°C, in the following step, the cells were fixed in 70% ethanol and stained with crystal violet (0.5% in ethanol). An Annexin V-FITC Apoptosis Detection Kit was used to determine the degree of apoptosis among the cells. (Beyotime). Three independent assays were performed for each assay.

### Quantitative RT–PCR (qRT–PCR)

The total RNA was extracted from tissues or cell lines of GBC using Trizol reagent (Invitrogen). In reverse transcription, cDNA was synthesized using the iScript™ cDNA synthesis kit (Bio-Rad). With the ABI Prism 7500 system (Applied Biosystems, CA) and SYBR Premix Ex Taq (Takara, Japan) using the 2^−∆∆ct^ method, real-time reverse transcription PCR was conducted to measure the expression levels of target genes. Normalization of expression results for genes was achieved using the internal control, β-actin. The primer sequences were as follows: TRIM37, forward: 5′TATGGAGAAATTGCGGGATGC 3′ and reverse: 5′GTCAGCCAGCGCCTAATACAG3′; Axin1, forward: 5′GACCTGGGGTATGAGCCTGA3′ and reverse: 5′GGCTTATCCCATCTTGGTCATC3′; β-Actin, forward: 5′CATGTACGTTGCTATCCAGGC3′ and reverse: 5′CTCCTTAATGTCACGCACGAT 3′.

### Western blot

Following the extraction of the total protein, it was separated by SDS-PAGE and then transferred to a polyvinylidene fluoride membrane. After that, we blocked the membrane with 5% non-fat milk in 0.1% PBST and incubated it with the indicated antibodies. The primary antibodies used were as follows: TRIM37(sc-49548, Santa Cruz Biotechnology), Axin1(#2074, CST), β-Catenin (#8480, CST), Active β-Catenin (#19807, CST), C-Myc (#9402, CST), Cyclin D1(#2922S, CST), β-Actin (A5441, Sigma). Afterward, A secondary antibody (A0208, A0216, Beyotime, China) conjugated to HRP was then used to probe the membranes. Finally, enhanced chemiluminescence (Bio-Rad) was used to visualize the membranes.

### Co-immunoprecipitation and ubiquitination assay

Lysates of cells were collected and immunoprecipitated overnight at 4°C with indicated or anti-IgG antibodies with gentle rotation. Over a two-hour period, we pulled down the antibodies using protein A/G-agarose beads (Santa Cruz). After extensive washing, immunocomplexes were resuspended in SDS loading buffer, followed by western blotting and IP assay. In the ubiquitination assay, Transfections with the HA-ubiquitin plasmid were performed on shTRIM37 stable transfected cells or on shControl cells. 48 hours after transfection, either two mg of HA-tag or IgG antibody was used to pull down the cell lysates. Separation of the immunoprecipitates by SDS-PAGE and immunoblotting with each antibody were performed.

### In vivo tumor xenograft model

In the present study, two randomly selected groups of female BALB/c nude mice (4-5 weeks old) were purchased from Shanghai Laboratory Animal Company. Inoculation of 1 × 10^6^ GBC-SD cells infected shTRIM37 or shControl was performed under the armpits by subcutaneous injection. The sizes of tumors were measured twice a week with calipers. Excision, weighing, and photography of the tumors were performed after the mice were sacrificed on the 21st day.

### Statistical analysis

Statistical analysis was conducted with SPSS 22.0 and GraphPad Prism 6 software. Student's t-tests were used to compare groups, while Pearson's correlation test was performed to analyze data relating TRIM37 expression to clinicopathological characteristics. As part of the survival analysis, the Kaplan-Meier method and the Log-Rank test were used. The prognostic factors that were independent have been identified using multivariate and univariate Cox proportional hazard regression models. P values of less than 0.05 were classified as statistically significant.

## Results

### TRIM37 expression is upregulated in GBC and correlated with poor prognosis

**Fig. 1:**
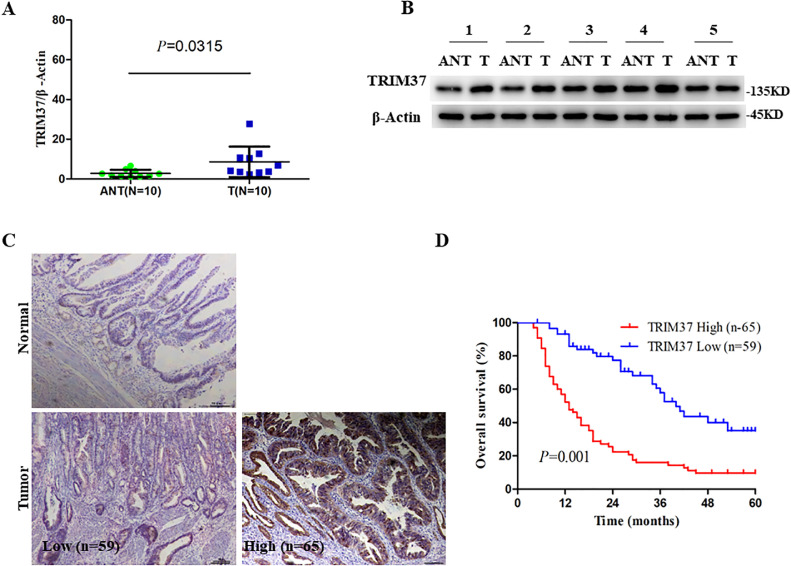
Overexpression of TRIM37 indicated poor prognosis of GBC patients. A. qRT-PCR analysis of TRIM37 expression in 10 pairs of GBC tissues and adjacent non-tumor tissues (ANT). B. TRIM37 expression is upregulated in GBC tissues analyzed by western blot. C. Representative IHC staining images of normal gallbladder tissues and GBC tissues with low expression and high expression of TRIM37. D. Kaplan-Meier analysis of the correlation between TRIM37 expression and the overall survival of GBC patients.

An examination of TRIM37 expression in clinical GBC tissue led us to speculate about its possible role in the disease. An analysis of 10 pairs of GBC and adjacent non-cancerous tissues performed using qRT-PCR revealed that the mRNA levels were higher in GBC than in adjacent non-cancerous tissues (Figure. 1A). Furthermore, western blot analysis revealed that normal control tissues had lower levels of TRIM37 protein than GBC tissues in 5 pairs of GBC tissues (Figure. 1B). The role of TRIM37 in GBC was assessed using immunohistochemistry (IHC) using paraffin-embedded, archived GBC tissue samples. A higher level of TRIM37 expression was detected in GBC tissues than in cholecystolithiasis tissues (Figure. 1C).

An evaluation of the associations between TRIM37 expression and clinicopathological characteristics was conducted to clarify the clinical significance of TRIM37 expression in GBC. Based on the results of the IHC staining, the 124 patients with GBC were grouped into a low expression TRIM37 group (59 patients) and a high expression TRIM37 group (65 patients). According to Table 1, high tumor differentiation (P=0.013) and advanced TNM stage (P=0.020) were positively associated with high TRIM37 expression. Furthermore, Kaplan-Meier analysis revealed that patients with high level TRIM37 expression had poorer overall survival (OS) (Figure. 1D). In summary, there is a strong correlation between upregulation of TRIM37 in GBC and poor clinical outcomes, which indicates that TRIM37 exerts oncogenic properties in GBC.

### TRIM37 promotes proliferation capabilities of GBC cells in vitro

**Fig. 2:**
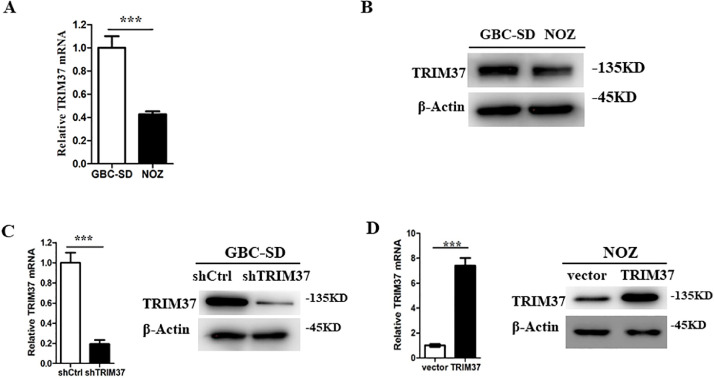
Expression level of TRIM37 was higher in GBC-SD cells. A. qRT-PCR analysis of TRIM37 expression in GBC-SD and NOZ cell lines. B. The protein levels of TRIM37 in two GBC cell lines. C, D. qRT-PCR (left) and western blot (right) analysis of TRIM37 expression in TRIM37-depleting GBC-SD cells and TRIM37-overexpressing NOZ cells. β-Actin was detected as an internal control.

To elucidate whether TRIM37 affected the biological characteristics of GBC, we examined two GBC cell lines (GBC-SD and NOZ) for TRIM37 expression as a first step. In GBC-SD cells, TRIM37 expression levels were higher than in NOZ cells according to qRT-PCR and western blot analysis (Figure. 2A and B). Following this, shRNA-lentivirus vectors targeting TRIM37 were used to stable transfect GBC-SD cells, and TRIM37 translation vectors were used to stabilize transfect NOZ cells. There was a considerable difference in efficiency between knockdown and overexpression of TRIM37 (Figure. 2C and D).

**Fig. 3:**
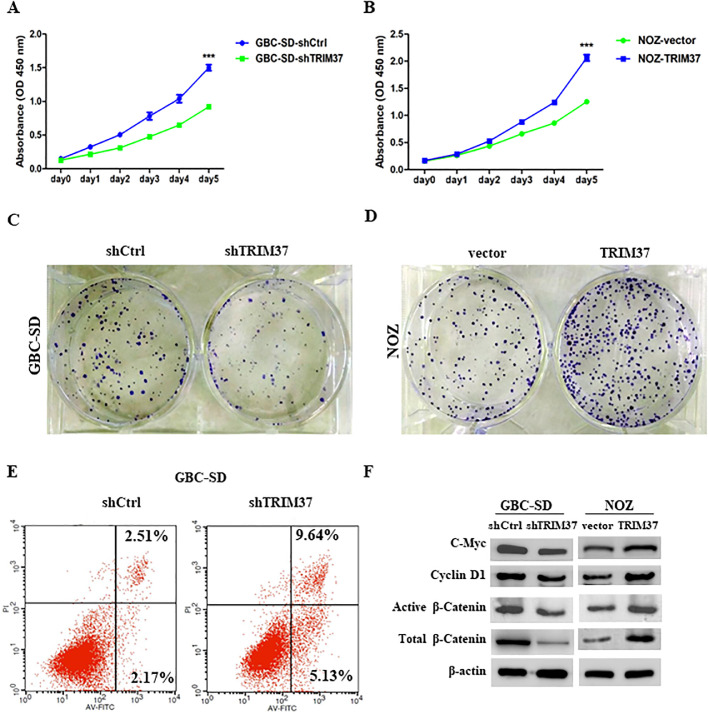
Effect of TRIM37 expression on proliferation, colony formation and apoptosis assay of GBC cells. A, B. CCK-8 assays were used to detect cell growth upon TRIM37 overexpression or knockdown. C, D. Colony formation assay was performed to examine the colony formation ability of GBC cells following shRNA knockdown and overexpression of TRIM37. E. Apoptosis was analyzed by flow cytometry in TRIM37 knockdown GBC-SD cells. F. The expression of β-catenin, active β-catenin, C-Myc and Cyclin D1 were examined by western blotting in GBC-SD and NOZ cells.

In order to determine whether TRIM37 affects the proliferation of GBC cells, CCK-8 and colony formation assays were performed. TRIM37 knockdown significantly reduced the proliferation of GBC-SD cells, whereas TRIM37 overexpression increased the proliferation of NOZ cells (Figure. 3A and B). Accordingly, a similar trend was also showed in colony formation experiments. In comparison with empty vector control cells, GBC-SD cells with TRIM37 knockdown showed reduced colony formation abilities; however, NOZ cells with TRIM37 overexpression showed increased colony formation abilities (Figure. 3C and D). Moreover, as a result of TRIM37 knockdown, both the early and late stages of cell apoptosis in GBC-SD were significantly increased by AnnexinV/PI staining (Figure. 3E).

As part of our investigation into a deeper understanding of the molecular mechanisms underlying the process, we explored the role of TRIM37 in the activation of the Wnt/β-catenin signaling pathway. As shown in Figure. 3F, western blot indicated the expression of total β-catenin and active β-catenin, along with two well-known targets of β-catenin, C-Myc and Cyclin D1, were decreased in TRIM37 knockdown GBC-SD cells. On the contrary, TRIM37 overexpression promoted the expression of β-catenin, C-Myc and Cyclin D1 (Figure. 3F).

### TRIM37 interacts with and induces ubiquitination of Axin1

**Fig. 4:**
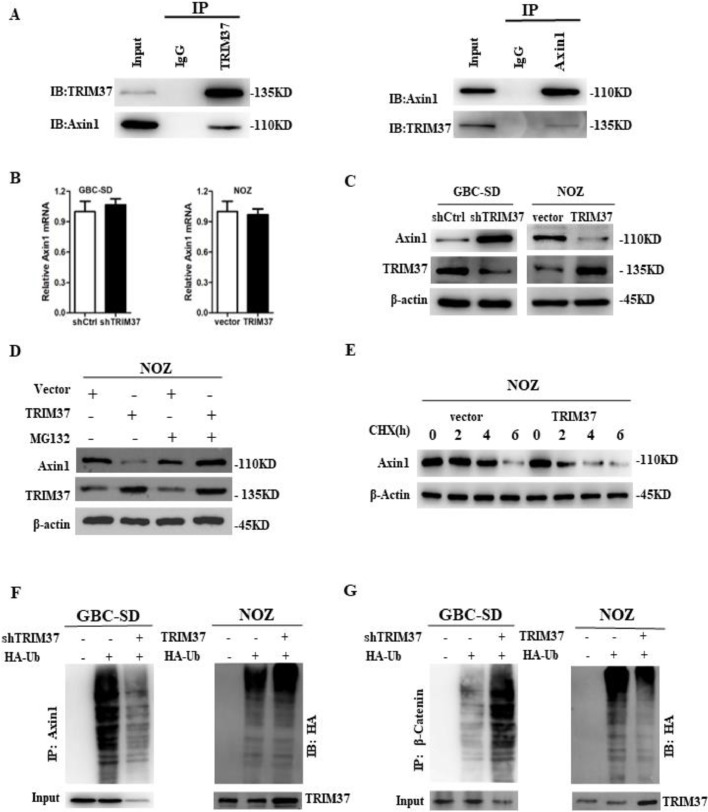
TRIM37 interacted with and promoted the ubiquitination of Axin1. A. The interaction between TRIM37 and Axin1 in GBC-SD cells was measured by coimmunoprecipitation. B. mRNA expression of Axin1 in GBC cells with TRIM37 knockdown and overexpression. C. The expression of TRIM37 and Axin1 and were examined by western blotting in GBC-SD and NOZ cells. D. Effects of protease inhibitor MG132 (10μM) on TRIM37-induced Axin1 expression. E. The overexpression of TRIM37 affected the half -life of Axin1. F, G. The ubiquitination of Axin1 and β-catenin was examined after transfection with HA-Ub and/or shTRIM37/ TRIM37.

The bioinformatic analysis of TRIM37 was performed using HitPredict (http://www.hitpredict.org/) to explore its molecular mechanism on regulating Wnt/β-catenin signaling pathway. The predicted substrates for TRIM37 include Axin1 which encodes a cytoplasmic protein that functions as a negative regulator of Wnt/β-catenin signaling, was studied in detail. As shown in Figure. 4A, The TRIM37 protein was found to coimmunoprecipitate with Axin1, and reciprocal immunoprecipitation with Axin1 antibodies reduced its level. These results indicate that TRIM37 interacts directly with Axin1.

Due to the fact that TRIM37 has a functional RING domain, which is associated with an E3-ubiquitin ligase, TRIM37 was hypothesized to regulate the protein level of Axin1. Figure 4B shows that TRIM37 knockdown or overexpression did not affect Axin1 mRNA levels. However, it did affect the expression of Axin1 protein. As TRIM37 was knocked down in GBC-SD cells, Axin1 protein expression increased, while it decreased in NOZ cells overexpressing TRIM37 (Figure. 4C). Furthermore, NOZ cells overexpressing TRIM37 could be treated with proteasome inhibitor MG132 to reverse the decreased Axin1 protein levels (Figure. 4D). Meanwhile, TRIM37 could reduce the half-life of the Axin1 protein after blocking translation using cycloheximide (CHX) treatment (Figure. 4E), which suggests that TRIM37 is responsible for regulating Axin1 expression through proteasome-dependent mechanisms. Ubiquitination of Axin1 is enhanced by TRIM37 overexpression while attenuated by TRIM37 knockdown (Figure. 4F). Consistently, knockdown of TRIM37 caused enhanced in β-catenin polyubiquitination, while TRIM37 overexpression reduced it (Figure. 4G). Based on these results, it appears that TRIM37 is responsible for activating Wnt/β-catenin signaling in GBC cells by degrading Axin1.

### Knockdown of TRIM37 inhibits tumor growth in vivo

**Fig. 5:**
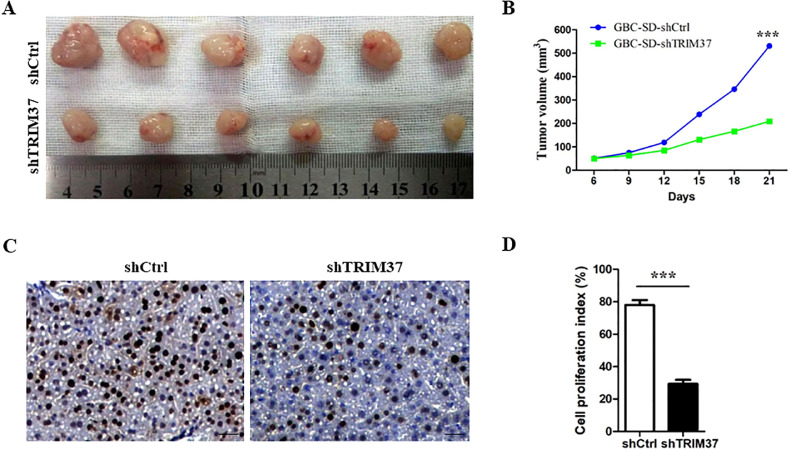
The knockdown of TRIM37 inhibits tumor growth in vivo. A. Xenografts transfected with shTRIM37 or shCtrl were photographed at the end of the experiment. B. Xenograft tumor volumes were measured every 3 days for 21 days using calipers. C. Cell proliferation in xenografts were determined by Ki-67 staining. D. Cell proliferation index was quantified by counting the proportion of nuclear Ki-67-positive cells. ***P < 0.001, compared with control shRNA group (Student's t-test).

In order to determine the effect of TRIM37 knockdown on GBC tumorigenesis, a xenograft mouse model was established using GBC-SD cells knocked down for TRIM37. The cells were subcutaneously injected into nude mice for 4 weeks, after which they were monitored. As shown in Figure. 5A and B, TRIM37 knockdown significantly inhibited xenograft tumor formation and growth. Additionally, IHC staining indicated that TRIM37 knockdown xenograft tumor tissue had a decreased expression of cell proliferation marker Ki-67 (Figure. 5C and D).

## Discussion

GBC is the most common cancer in the biliary tract characterized by high mortality rates, late diagnosis and poor clinical presentation [14,15]. Despite advances in GBC therapy, the prognosis for most patients remains dismal. Consequently, further studies are necessary to identify novel molecular markers and therapeutic targets for prognostic and therapeutic considerations in GBC [16]. Currently, growing evidence suggests that TRIM37 is an oncogene and may serve as a potential therapeutic target in human cancers. Several types of cancer show high TRIM37 expression. The present study reveals the critical role of TRIM37 in GBC and its underlying mechanisms. Furthermore, we confirm that TRIM37 is an important prognostic indicator for patients with GBC.

This study explores for the first time the expression level and biological function of TRIM37 in GBC. The expression of TRIM37 was significantly higher in GBC tissues compared with peritumoral tissues, and its high expression was significantly associated with poor tumor differentiation, along with advanced TNM stage and a shorter OS of GBC patients. According to these findings, TRIM37 may play an important role in the progression of GBC, and might be a novel prognostic factor for patients with this disease. In this study, we demonstrated that knockdown of TRIM37 suppresses proliferation and enhances apoptosis in GBC cells. Conversely, overexpression of TRIM37 promotes GBC cell proliferation. Additionally, Nude mice with TRIM37 knockdown show decreased tumor growth in vivo.

This study also revealed a novel finding, which is that TRIM37 promotes GBC development by activating Wnt/β-catenin signaling through the degradation of Axin1. It has been reported recently that aberrant Wnt/β-catenin signaling may contribute to the development of GBC [17]. Several studies have demonstrated that TRIM37 is involved in aberrant activation of the Wnt/β-catenin signaling pathway. In GBC cells, TRIM37 has been found to interact with Wnt/β‑catenin signaling pathway, although the mechanisms behind this remain unknown [11,12,[18], [19], [20], [21]]. It has been shown in previous studies that the E3 ubiquitin ligases, such as RNF146, TRIM11, TRIM32, TRIM54, TRIM65 and UBE3C promote various cancers by triggering Wnt/β-catenin signaling via degradation of Axin1 [22], [23], [24], [25], [26], [27]. Axin1 and GSK-3β are two key regulators of the Wnt/β-Catenin pathway. Axin1 promotes GSK-3β-mediated phosphorylation and degradation of β-catenin by interacting with APC, GSK-3β and β-catenin [27]. Despite this, there is still no clear understanding of the molecular mechanism controlling Axin1 and β-catenin signaling in GBC. We postulated TRIM37 might have an important role in Wnt/β-catenin signaling through its effects on GBC cells. In our study, TRIM37 affected β-catenin, cyclin D1, and C-Myc expression of GBC cells. Here, the Co-IP experiments confirmed the interaction between TRIM37 and Axin1. Furthermore, TRIM37 knockdown led to a reduction in ubiquitination of Axin1, which led to β-catenin degradation.

The results of our study provide the first evidence that the TRIM37 gene is clinically and biologically relevant to GBCs. Our findings indicate that TRIM37 promotes GBC development via activation of Wnt/β-catenin signaling via ubiquitination and degradation of Axin1. As a result of this study, we have identified a molecular mechanism that drives GBC progression and have also uncovered a potential biomarker for diagnosis, as well as new insights into treatment and prognosis of GBC patients.

## Authors’ contributions

M.X. preformed experiments and data analyses, wrote original draft. B.J. performed experiments and data analyses. Z.M. and H.Z. conceived and supervised the project, reviewed and edited the manuscript. H.Z. made graphical abstract by using Figuredraw software.

## Funding

This study was supported by the Key Project of Education Department of Anhui Province (KJ2020A0562), 10.13039/501100003995Natural Science Foundation of Anhui Province in China (2108085QH342) and Science Fund for Outstanding Young Scholars of the First Affiliated Hospital of Bengbu Medical College (2021byyfyyq03).

## Declaration of Competing Interest

There are no conflicts of interest reported by the authors.

## References

[1] Koppatz H, Takala S, Peltola K, But A, Mäkisalo H, Nordin A (2021). Gallbladder cancer epidemiology, treatment and survival in Southern Finland - a population-based study. Scand. J. Gastroenterol..

[2] Vega EA, Mellado S, Salehi O, Freeman R, Conrad C. (2022). Treatment of resectable gallbladder cancer. Cancers.

[3] Kamada Y, Hori T, Yamamoto H, Harada H, Yamamoto M, Yamada M (2020). Surgical treatment of gallbladder cancer: An eight-year experience in a single center. World J. Hepatol..

[4] Park Y, Kim K, Park HJ, Chun HJ, Choi D, Kim K. (2022). Role of adjuvant treatment in high-risk patients following resection for gallbladder cancer. In Vivo.

[5] Neogi K, Tewari M, Singh AK, Sharma K, Tej G, Verma SS (2022). Transcription factor 4 expression and correlation with tumor progression in gallbladder cancer. J. Cancer Res. Therapeut..

[6] Liang C, Yang P, Han T, Wang RY, Xing XL, Si AF (2019). Long non-coding RNA DILC promotes the progression of gallbladder carcinoma. Gene.

[7] Liu X, Zuo X, Sun X, Tian X, Teng Y. (2022). Hexokinase 2 promotes cell proliferation and tumor formation through the Wnt/β-catenin pathway-mediated cyclin D1/c-myc upregulation in epithelial ovarian cancer. J. Cancer.

[8] Kim JY, Park G, Krishnan M, Ha E, Chun KS. (2019). Selective Wnt/β-catenin small-molecule inhibitor CWP232228 impairs tumor growth of colon cancer. Anticancer Res..

[9] Brigant B, Metzinger-Le Meuth V, Rochette J, Metzinger L (2018). TRIMming down to TRIM37: relevance to inflammation, cardiovascular disorders, and cancer in MULIBREY nanism. Int. J. Mol. Sci..

[10] Li D, Zhang Z. (2022). TRIM37 promotes the aggressiveness of ovarian cancer cells and increases c-Myc expression by binding to HUWE1. Arch. Biochem. Biophys..

[11] Jiang J, Tian S, Yu C, Chen M, Sun C. (2016). TRIM37 promoted the growth and migration of the pancreatic cancer cells. Tumour Biol..

[12] Jiang J, Yu C, Chen M, Tian S, Sun C. (2015). Over-expression of TRIM37 promotes cell migration and metastasis in hepatocellular carcinoma by activating Wnt/β-catenin signaling. Biochem. Biophys. Res. Commun..

[13] Zhu H, Chen Y, Zhang J, Qian C, Qiu W, Shen H (2020). Knockdown of TRIM37 promotes apoptosis and suppresses tumor growth in gastric cancer by inactivation of the ERK1/2 pathway. OncoTargets Ther..

[14] Halaseh SA, Halaseh S, Shakman R. (2022). A review of the etiology and epidemiology of gallbladder cancer: what you need to know. Cureus.

[15] Ramalhosa F, Amaral MJ, Serôdio M, Oliveira RC, Teixeira P, Cipriano MA (2022). Clinicopathological prognostic factors for gallbladder carcinoma: a retrospective study. J. Gastrointestinal Oncol..

[16] Yu H, Xu Y, Gao W, Li M, He J, Deng X (2022). Comprehensive germline and somatic genomic profiles of Chinese patients with biliary tract cancer. Front. Oncol..

[17] Dixit R, Pandey M, Tripathi SK, Dwivedi AND, Shukla VK. (2020). Genetic mutational analysis of β-catenin gene affecting GSK-3β phosphorylation plays a role in gallbladder carcinogenesis: Results from a case control study. Cancer Treatm. Res. Commun..

[18] Li D, Zhang Z. (2022). TRIM37 promotes the aggressiveness of ovarian cancer cells and increases c-Myc expression by binding to HUWE1. Arch. Biochem. Biophys..

[19] Ding Y, Lu Y, Xie X, Sheng B, Wang Z. (2018). Silencing TRIM37 inhibits the proliferation and migration of non-small cell lung cancer cells. RSC Adv..

[20] Zhao P, Guan HT, Dai ZJ, Ma YG, Liu XX, Wang XJ. (2017). Knockdown of tripartite motif-containing protein 37 (TRIM37) inhibits the proliferation and tumorigenesis in colorectal cancer cells. Oncol. Res..

[21] Tao Y, Xin M, Cheng H, Huang Z, Hu T, Zhang T (2017). TRIM37 promotes tumor cell proliferation and drug resistance in pediatric osteosarcoma. Oncol. Lett..

[22] Zhou L, Wang H, Zhong M, Fang Z, Le Y, Nie F (2022). The E3 ubiquitin ligase TRIM11 facilitates gastric cancer progression by activating the Wnt/β-catenin pathway via destabilizing axin1 protein. J. Oncol..

[23] Zhu J, Wu Y, Lao S, Shen J, Yu Y, Fang C (2021). Targeting TRIM54/Axin1/β-catenin axis prohibits proliferation and metastasis in hepatocellular carcinoma. Front. Oncol..

[24] Zhang Y, Xu J, Fu H, Wei Z, Yang D, Yan R. (2021). UBE3C promotes proliferation and inhibits apoptosis by activating the β-catenin signaling via degradation of AXIN1 in gastric cancer. Carcinogenesis.

[25] Chen F, Guo Q, Chen Q, Han Z, Zhou X, Wu L (2020). TRIM32 triggers β-catenin signaling through ubiquitylation of AXIN1 to promote inflammatory factor-induced apoptosis of rat nucleus pulposus cells. Am. J. Physiol. Cell Physiol..

[26] Shen J, Yu Z, Li N. (2018). The E3 ubiquitin ligase RNF146 promotes colorectal cancer by activating the Wnt/β-catenin pathway via ubiquitination of Axin1. Biochem. Biophys. Res. Commun..

[27] Yang YF, Zhang MF, Tian QH, Zhang CZ. (2017). TRIM65 triggers β-catenin signaling via ubiquitylation of Axin1 to promote hepatocellular carcinoma. J. Cell Sci..

